# Subtypes of Batterers in Treatment: Empirical Support for a Distinction between Type I, Type II and Type III

**DOI:** 10.1371/journal.pone.0110651

**Published:** 2014-10-16

**Authors:** José Luis Graña, Natalia Redondo, Marina J. Muñoz-Rivas, Arthur L. Cantos

**Affiliations:** 1 Faculty of Psychology, Complutense University of Madrid, Madrid, Spain; 2 Faculty of Psychology, Autónoma University of Madrid, Madrid, Spain; 3 Department of Psychology, Rosalind Franklin University of Medicine and Science, Chicago, Illinois, United States of America; VIB & Katholieke Universiteit Leuven, Belgium

## Abstract

This study explores the existence of different types of batterers in a sample of 266 men who had been court referred for intimate partner violence. The data collected in the assessment that have been used to perform a hierarchical and a two-step cluster analysis fall into three areas: aggression towards the partner, general aggression and presence of psychopathology and personality traits, more specifically, alcohol use, borderline and antisocial personality traits, psychopathy traits, state anger and trait anger, anger expression and control, anger, hostility, and, finally, impulsivity. The results show a typology consisting of 3 types of batterers on the basis of violence level and psychopathology: low (65%), moderate (27.8%) and high (7.1%). This study provides empirical support for the development of batterer typologies. These typologies will help achieve early detection of different types of batterers, allowing us to tailor interventions on the basis of the needs of each of the types.

## Introduction

Beginning in the 1980's, domestic violence researchers attempted to move beyond theoretical conjecture in order to empirically describe the characteristics of men who perpetrate violence against their intimate partners. Initial efforts were focused on finding the common elements that would differentiate perpetrators from non-perpetrators [1]. It quickly became apparent that there is no “unitary batterer profile in terms of personality, psychopathology, or demographics” [2]. Since perpetrators form a heterogeneous group, most recent efforts have been directed towards identifying meaningful perpetrator subtypes (i.e., identifying commonalities that differentiate subgroups of perpetrators from one another and from non-perpetrators).

To date, the most important study on types of perpetrators is the one by Holtzworth-Munroe and Stuart [3], due to the relevance of its approach and the subsequent empirical support it has received. Holtzworth-Munroe and Stuart [3] reviewed existing male perpetrator typologies to determine the subtypes that consistently appeared across typological models and identified three major dimensions that have been used to distinguish among subtypes. These dimensions were (a) the severity of marital physical violence and related abuse, such as frequency of the violence and psychological and sexual abuse; (b) the generality of violence (i.e., family-only or extrafamilial violence) and related variables such as criminal behavior and legal involvement; and (c) the perpetrator's ’s psychopathology or personality disorders. They further hypothesized that researchers using these three descriptive dimensions would usually identify three major subtypes of batterers: family only, dysphoric/borderline, and generally violent/antisocial.

On the basis of Holtzworth-Munroe and Stuart [3] findings, different authors have attempted to replicate their typology. Hamberger et al. [4] attempted to empirically validate the Holtzworth-Munroe and Stuart [3] typology with 204 court referred men. They found three groups of perpetrators which were similar to Holtzworth-Munroe and Stuart [3] groups: a low-risk non pathological group, with low levels of violence occurring in the home only, similar to Holtzworth-Munroe and Stuart [3] family-only batterer group. A passive aggressive-dependent group similar to the dysphoric-borderline personality group, displaying attachment problems and mental health problems, and anantisocial group mirroring the generally violent/antisocial group with increased violence that extends outside intimate relationships.

Holtzworth-Munroe et al. [5] also attempted to empirically validate their 1994 model with a community sample of 102 men who were aggressive towards their partners. Utilizing the three descriptive dimensions of severity of marital violence, generality of violence and psychopathology, four clusters of perpetrators were identified. Three resembled the predicted subtypes, of family-only men (who resembled nonviolent controls, except for their violent behavior), generally violent/antisocial men, and a borderline/dysphoric group. The final cluster, given their intermediate scores on most scales and their higher score on the antisociality scale, was named the low-level antisocial abuser. Holtzworth-Munroe et al. [5] stated that this group is probably analogous to the originally proposed family-only group and other family-only types identified in previous typologies. Holtzworth-Munroe et al. [5] referred to Hamberger et al. [4] nonpathological group as closely resembling the low-level antisocial abuser on several measures such as mean level of husband violence and mean number of arrests.

In a more recent study with 671 court referred men, Stoops et al. [6], after conducting a cluster analysis with information from the three dimensions proposed by Holtzworth-Munroe and Stuart [3], were able to replicate the finding of three types of batterers and classified them into the following groups: low level criminality, dysphoric volatile behavior and dysphoric general violence. The most novel aspect of this study was their analysis of the levels of recidivism and the finding that the typology predicted both program completion and re-arrest. However, contrary to Holtzworth-Munroe and Stuart [3] predictions, they found that the low level criminality group had higher levels of aggression towards the partner and of general violence than the dysphoric volatile behavior group. Stoops et al. [6] argue that the inconsistency in findings could be explained by the fact that their sample consisted of court referred men, whereas Holtzworth-Munroe et al. [5] sample consisted of community violent men with lower levels of aggression. Stoops et al. [6], further hypothesize that the low level criminality group would present similar characteristics to the low-level antisocial group proposed by Holtzworth-Munroe et al. [5].

A system for categorizing partner-violent men as either reactive or proactive aggressors was developed and evaluated by Chase et al. [7]. Sixty partner-violent men were reliably categorized, and the distribution (62% reactive, 38% proactive) fell within the expected range. Some construct validity was demonstrated, as several significant predicted group differences were found on factors of theoretical relevance to the typology model (affectivity, personality, and violence in the family-of-origin). Proactively versus reactively categorized participants were (a) more dominant and less angry during a 10-min interpartner interaction, (b) more antisocial and aggressive-sadistic and less dependent, and (c) more frequently classified as psychopathic (17% vs. 0%). Research and clinical implications of the system are discussed, as is the potential overlap between the reactively and proactively categorized partner-violent men in this study with previously identified types [7].

While there has been widespread interest in the topic, efforts to replicate the three part batterer typology of Holtzworth-Munroe and Stuart [3] have met with mixed success [4], [5], [8], [9]. Boyle et al. [10] suggested that a more easily applied method of distinguishing between subgroups of partner violent men, based on a theoretically important behavioral distinction (i.e., the generality of the violence committed) provides a better focus for research in this area and found that generally violent and partner only violent men differed on a number of characteristics including lifetime history of conduct disordered and delinquent behavior, behavioral disinhibition, lifetime psychological abuse of intimate partners and family of origin violence.

The results obtained regarding batterer typologies have thus provided evidence that supports heterogeneity in the characteristics of male perpetrators of intimate partner violence in both community and court mandated samples. They have also show that using a variety of typing techniques, most of the typology studies found continued support for Holtzworth-Munroe and Stuart [3] tri-fold typology.

In keeping with the approach emphasizing the heterogeneity of perpetrators and of the violence itself, some authors have explored another dimension of heterogeneity and have argued that what is relevant in establishing the typologies is to consider the risk of abuse and the likelihood of re-offense, rather than the individual characteristics of each type. Cavanaugh and Gelles [11] compiled the most common batterer typologies [3], [4], [12]–[14] and successfully identified similarities across models. They found most typologies categorized batterers along a continuum in which each subtype generally indicated low, moderate or high risk. Low-risk batterers generally had low severity of violence, low frequency of abuse, little psychopathology and no criminal history. Moderate-risk subtypes included batterers with moderate abuse severity and frequency and moderate to high psychopathology. Finally, high-risk subtypes across typologies generally described batterers who had high violence severity, frequent battering incidents, psychopathology and other forms of criminal behavior [11].

The aim of this research is to examine whether in a sample of court referred men in Spain we can find these 3 types of perpetrators of intimate partner violence on the basis of violence level and presence of psychopathology. The information used to establish the typology in this study will be the frequency and severity of violence towards the partner and general aggression levels, as well as factors that have often been associated with risk of abusing the partner, in particular, risk factors belonging to 3 areas: personality characteristics, alcohol use, impulsivity and violence-related emotions.

## Method

### Ethics Statement

The study was approved by the Bioethics Committee of the Faculty of Psychology of the Complutense University of Madrid the 30^th^ of May of 2009. Written informed consent was obtained from all participants. All patients were informed of the purpose of the research, the estimation duration of the treatment and the procedure that were followed for the completion of the research.

### Participants

The patients who participated in this study were court referred men from the Region of Madrid who have been convicted of a crime of gender violence placed in mandated court diversion program. They are required to attend a psychological treatment program in lieu of completing a prison sentence of less than two years as provided for in Title IV of Organic Act 1/2004 [15], whose Article 35, regarding penalties substitution, indicates that: *“Where an offender has been sentenced for a crime related to gender violence, […] the Judge or Court shall order the offender’s […] attendance at specific re-education and psychological therapy courses.”*

Exclusion criteria for involvement in the treatment program at which these data were collected included reports of current drug abuse, current heavy drinking (six or more drinks per day) or the presence of acute psychotic symptoms. Men who did not meet any treatment program exclusion criteria, and who could read, write, and speak in Spanish were asked to participate in this study.

The total study sample consisted of 266 men aged between 18 and 69 years, with a mean age of 37.78 (*SD* = 10.09). Ninety per cent were convicted of physical violence −the most frequent forms being frequent hitting, grabbing, hair pulling and shaking−, whereas 10.2% were convicted of psychological violence −mainly threatening and insulting their partners−. Forty one per cent had completed elementary school, 41% had completed high school and 18% had attended some college at least. Twenty four percent (24.4) were married, 2.3% were remarried, 4% were widowed, 15% were separated, 18.8% were divorced, 34.6% were single and, finally, 4.5% were cohabitating as common-law partners. Around half of the sample were Spanish (53%), 38% were from Latin American countries and 9% from other countries.

### Procedure

Participants attended a series of sessions with two Masters-degree level therapists. The entire assessment phase was conducted individually and consisted of 4 to 8 weekly sessions of 60 minutes in which the following clinical activities were carried out:

a) During the first session, the lead therapist explained the study in detail, and obtained the participant’s informed consent to participate in the present study, which involved confidential self-report assessments.

b) After signing the informed consent, the therapist collected, by means of an interview, a series of socio-demographic data relating to the couple and the offense for which the male partner has been convicted.

c) A series of additional measures − which are described in the *Measures* section− were administered. The instructions were read aloud to the patient, and help was provided with completion of the first item of each instrument clarifying any doubts that might arise. The patient was given time to answer each of the tests, using as many sessions as necessary for the completion of the tests. In order to minimize socially desirable responding, participants were informed that therapists leading the groups would be blind to their responses. All questionnaires were self-report, and all questions about the relationship referred to the relationship leading to domestic violence charges (i.e., not necessarily the relationship at the time of questionnaire completion).

d) Motivational enhancement procedures were used during the assessment phase with the goal of increasing treatment compliance with particular emphasis on the benefits that could be obtained with the completion of the treatment program, such as complying with the law, knowing the way they relate and the function of aggression in intimate relationships.

### Measures

#### Sociodemographic Questionnaire

Diverse items were included to assess participants' characteristics in the following sociodemographic and personal variables: age, civil status, nationality, studies and professional activity. The information relating to the crime was obtained through the analysis of court sentences. Victims information was no included as there was no permission to do so.

#### Severity-frequency of violence towards the partner

This dimension was measured with the *Revised Conflict Tactics Scale* (*CTS2*; [16], Spanish adaptation of Loinaz et al. [17]). The CTS2 is a 78-item self-report questionnaire assessing behaviors during relationship conflict. Thirty nine items ask about perpetration and 39 items ask about victimization within the past year, across five subscales (negotiation, psychological aggression, physical assault, injury, sexual coercion). According to Straus et al. [16], the coefficient *alpha* ranged from.79 to.95 for subscales of the CTS2. In the present study, *alpha* for the full perpetration scale was.83.

#### Alcohol use

The evaluation of alcohol use and dependence was measured with two tools. *Alcohol Use Disorders Identification Test* (*AUDIT*; [18]) is a 10-item measure of alcohol consumption, dependence, and related consequences that is widely used for both research and clinical purposes. It has good internal consistency and excellent sensitivity and specificity as an alcohol screen [19]. In the present study, internal consistency was adequate (α = .77). *CAGE Questionnaire* [20] is an alcoholism screening tool widely used in clinical settings outside addiction treatment. It consists of 4 items with true-false answers. The first 3 items explore the person’s subjective aspects regarding alcohol use, while the last one assesses aspects concerning alcohol dependence. In the present study, *alpha* was.74.

#### Borderline and antisocial personality characteristics

In order to measure both dimensions, the *Self-report Assessment of the DSM-IV R Personality Disorders* (*SCID-II*; [21]) was used. It assesses the presence or absence of the symptoms included in the DSM IV for different personality disorders. In this study, only the items related to the borderline and antisocial personality scales (15 items for each scale) were administered. The authors point out that the test-retest reliability is.84 for the antisocial disorder and.37 for the borderline disorder. In this study, the reliability calculated using the coefficient *alpha* was.70 for the antisocial scale and.83 for the borderline scale.

#### Psychopathy characteristics

*Levenson Primary and Secondary Psychopathy Scale* (*LPSP*; [22]) was used. This 26 item self-report scale measures the domains of manipulation and callousness (primary psychopathy) and impulsivity (secondary psychopathy). It has adequate psychometric qualities in independent research [23]. This measure is viewed as an acceptable and appropriate self-report measure of psychopathic traits [24]. In the present study, *alpha* for the full scale was.77; *alpha* for the primary subscale was.69 and *alpha* for the secondary subscale was.63.

#### General aggression and aggression-related emotions

*Aggression Questionnaire* (AQ, [25], Spanish adaptation by Andreu et al. [26]) was used to measure general aggression. This 29-item questionnaire measures physical aggression, verbal aggression, anger and hostility. Test-retest reliability, correlations range from.72 for the anger subscale to.80 for the physical aggression subscale [25]. In the Spanish adaptation, the coefficient *alpha* was.86 for physical aggression, .77 for anger, .68 for verbal aggression and.72 for hostility [26]. In this study, the *alpha* reliability coefficient was.77 for physical aggression, .69 for verbal aggression, .79 for anger and.79 for hostility. *State-Trait Anger Expression Inventory* (STAXI-2; [27], Spanish adaptation by Miguel-Tobal et al. [28]), consisting of 49 items that measure state anger, trait anger and different forms of anger expression and control. The results found in all scales and subscales of the STAXI-2 indicate good internal consistency, with values ranging from.82 for *trait anger* to.69 and.67 for *anger expression* [28]. In this study, the *alpha* reliability coefficient was.92 for the state anger scale, .86 for the trait anger scale and.74 for the anger expression and control scale.

#### Impulsivity characteristics

To assess impulsivity traits, the *Barratt Impulsiveness Scale* [29] was used. It consists of 30 items and 3 subscales: *cognitive impulsiveness*, *motor impulsiveness* and *non-planning impulsiveness*. This scale has high internal consistency, between.89 and.92 [29], whereas in this study the coefficient *alpha* was.79.

### Statistical Analysis

According to Aldenderfer and Blashfield [30] five basic types of information should be reported when using cluster analysis: the computer program, the similarity measure, the cluster method, the procedure used to determine the number of clusters and the evidence for the validity of the clusters.

All statistical analyses were performed using the statistical software *SPSS 15.0*. First, reliability rates were determined through Cronbach’s coefficient *alpha* for each of the scales used in the study. To achieve the general goal of this investigation, two cluster analyses were performed: A hierarchical cluster analysis was performed first, in order to identify the statiscally most appropriate number of clusters [31] using Ward’s method of agglomerative clustering (using *Z*-scores), and squared Euclidean distances were used as a measure of similarity of cases. Hierarchical cluster analysis included the following variables specified in the *Measures* section: physical aggression, verbal aggression, anger, hostility, alcohol use (two measures), borderline and antisocial personality, primary and secondary psychopathy, impulsiveness, state anger, trait anger, anger expression and control, minor and severe psychological aggression, minor and severe physical aggression, minor and severe sexual coercion, minor and severe injury.

After having identified the most appropriate number of clusters, a two-step cluster analysis was performed (with all variables included in the hierarchical analysis and in the same order). All the variables were previously standardized. Bayesian clustering criterion of Schwarz (BIC) was used, and the measure of distance was the log-likelihood. This was followed by an ANOVA with post-hoc comparisons (Bonferroni) to identify significant differences between clusters in quantitative variables and a Pearson’s *chi-squared* test for qualitative variables, in particular, socio-demographic characteristics (level of education, occupation, marital status, nationality), type of partner at the time of the pre-treatment assessment, and the crime for which they were convicted. In order to validate the obtained clusters, an ANOVA with post-hoc comparisons (Bonferroni) was performed with several validation variables which were independent from the cluster analysis: police arrests for intimate partner violence, social and family problems, and drug use. Finally, to show that the clusters are stable, the two-step cluster analysis was repeated in a different randomly drawn sample from the same population.

## Results

Hierarchical cluster analysis suggested a three cluster solution. Analyzing amalgamation coefficients between cases through the agglomeration schedule, two inconsistent increases in the dissimilarity measure were observed, indicating that two and three clusters solutions might represent the data well. However, analyzing the dendrogram, in which scores are standardized in a 25 point scale (25 being the greatest distance between cases), it looks like a three cluster solution is the one that best reflects the underlying structure of the data, since three groups of relatively homogenous data without large amalgamation coefficients are observed. The four cluster solution was rejected because one of the four clusters contained less than 1% of the sample. The two cluster solution consisted of one group which contained 30.5% of the sample and another group which contained 69.5%; lastly, the three cluster solution is made up of three groups: 4.9%, 25.6% and 69.5% of the sample respectively.

Next, a two-step cluster analysis including the same variables and in the same order as in the hierarchical analysis was carried out to validate and verify the stability of the obtained solution. The two-step cluster analysis confirmed the existence of 3 groups of men with distinct psychological characteristics: a group of 19 patients, constituting 7.1% of the sample (referred to as Type I or high-level of aggression against the partner); a group of 74 patients, constituting 27.8% (Type II or moderate-level of aggression against the partner); and a third group of 173 patients, constituting 65% (Type III or low-level of aggression against the partner).

In analyses of the socio-demographic characteristics of the 3 groups: Statistically significant differences were found in relation to age (*p*<.05), as the mean age of Type III (39.03 years) is significantly higher than that of Type II (35.09 years) but no differences were found between Type I and the two others. Table 1 shows the differences between the three types in terms of socio-demographic characteristics (level of education, occupation, nationality, marital status), as well as the type of crime for which they had been convicted (physical or psychological violence) and the partner they had at the time of the assessment (the same partner that reported them, a new partner or no stable partner).

**Table 1:** Distribution of socio-demographic variables on the basis of group membership.

	Type I (n = 19)	Type II (n = 74)	Type III (n = 173)	*F*_(2,263)_/*χ2* Bonferroni
Age (years)	36.89±11.93	35.09±9.71	39.03±9.86	**4.11** * **2<3** *
Spanish	57.9% (A.R. = 0.4)	50% (A.R. = −0.6)	53.8% (A.R. = 0.3)	
South American	42.1% (A.R. = 0.4)	44.6% (A.R. = 1.4)	34.7% (A.R. = −1.5)	5.69^a^
Other nationalities	0% (A.R. = −1.4)	5.4% (A.R. = −1.3)	11.6% (A.R. = 2)	
Elementary studies	57.9% (A.R. = 1.6)	41.9% (A.R. = 0.2)	38.7% (A.R. = −1)	
High school	36.8% (A.R. = −0.4)	43.2% (A.R. = 0.5)	40.5% (A.R. = −0.2)	4.61^a^
College	5.3% (A.R. = −1.5)	14.9% (A.R. = −0.8)	20.8% (A.R. = 1.6)	
Managers/Businessmen/Civil servants/Office workers	0% (A.R. = −1.9)	9.5% (A.R. = −1.6)	**19.1% (A.R. = 2.5)**	
Unemployed people/Pensioners/Retired	**36.8% (A.R. = 3.1)**	12.2% (A.R. = −0.4)	11.6% (A.R. = −1.3)	15.28**^a^
Building industry/Hospitality/Industry	63.2% (A.R. = −0.8)	78.4% (A.R. = 1.6)	69.4% (A.R. = −1)	
Married/Common-law partners	36.8% (A.R. = 0.6)	29.7% (A.R. = −0.3)	31.2% (A.R. = 0)	
Single	31.6% (A.R. = −0.3)	41.9% (A.R. = 1.6)	31.8% (A.R. = −1.3)	2.99^a^
Widowed/Separated/Divorced	31.6% (A.R. = −0.3)	28.4% (A.R. = −1.2)	37% (A.R. = 1.3)	
The same partner that reported	0% (A.R. = −1.9)	16.2% (A.R. = 0.4)	15.6% (A.R. = 0.6)	
A different partner	52.6% (A.R. = 0.2)	56.8% (A.R. = 1.3)	47.4% (A.R. = −1.3)	6.31^a^
No partner	47.4% (A.R. = 1.2)	27% (A.R. = −1.7)	37% (A.R. = 0.9)	
Physical offense	100% (A.R. = 1.5)	89.2% (A.R. = −0.2)	89% (A.R. = −0.6)	
Psychological offense	0% (A.R. = −1.5)	10.8% (A.R. = 0.2)	11% (A.R. = 0.6)	2.31^b^

Note. Data refer to the mean ± standard deviation (*SD*) except those that refer to percentages.

*p<0.05 **p<0.01 ***p<001. A.R.  =  Adjusted residuals.

a *df* = 4,

b *df* = 2.

The results of Pearson’s *chi-squared* test indicated that there are significant differences only in two levels of the variable occupation. Specifically, it was found that for Type III the proportion of managers/businessmen/civil servants/office workers was higher than for the other two, while for Type I there was a higher proportion of unemployed people/pensioners/retirees than for Type II and Type III (*χ2* = 15.28, *p*<.01) (see Table 1).

Therefore, except in relation to occupation and age, the three groups are similar with respect to socio-demographic characteristics, type of crime and partner at the beginning of the psychological treatment program (Table 1).

Tables 2 and 3 show the means of each of the groups for all the variables used. Specifically, Table 2 shows the results of the variables referring to two of the dimensions proposed by Holtzworth-Munroe and Stuart [3]: generality of violence (general physical and verbal aggression) and presence of psychopathology (anger, hostility, alcohol use, borderline and antisocial personality characteristics, primary and secondary psychopathy traits, impulsivity, state anger, trait anger, anger expression and control). Table 3 shows the levels of aggression towards the partner for each of the 3 groups.

**Table 2:** Differences between the three types of batterers in variables related to generality of violence and presence of psychopathology.

	Sample (n = 266)	Type I High-level (n = 19)	Type II Moderate-level (n = 74)	Type III Low-level (n = 173)	*F*_(2,263)_	Bonferroni
**GENERAL AGGRESSION**						
AQ-Physical aggression	1.86±0.66	2.53±0.84	2.33±0.62	1.59±0.47	62.22***	1>3***; 2>3***
AQ-Verbal Aggression	2.17±0.77	2.96±0.78	2.69±0.70	1.86±0.61	57.38***	1>3***; 2>3***
AQ-Anger	2±0.78	2.95±0.72	2.67±0.72	1.61±0.46	120.63***	1>3***; 2>3***
AQ-Hostility	2.37±0.81	3.11±0.85	2.83±0.78	2.10±0.68	37.24***	1>3***; 2>3***
**ALCOHOL USE**						
AUDIT	5.73±4.74	11.90±7.11	6.95±5.17	4.53±3.43	29.14***	1>2***; 1>3***; 2>3***
CAGE	0.91±1.10	1.85±1.38	1.14±1.25	0.71±0.91	12.51***	**1>2** * ; 1>3***; 2>3 *
**PERSONALITY**						
SCID II-Borderline	3.91±2.85	8.05±3.43	5.50± 3	2.78±1.79	60.02***	1>2***; 1>3***; 2>3***
SCIDII-Antisocial	1.28±1.48	3.57±3.04	1.72±1.40	0.84±0.86	44.34***	1>2***; 1>3***; 2>3***
**PSYCHOPATHY**						
Levenson-primary	11.99±5.17	18.51±6.59	12.95±4.25	10.86±4.75	24.09***	1>2***; 1>3***; 2>3**
Levenson-secondary	8.04±3.89	14.59±4.45	9.73±3.43	6.59±2.86	69.50***	1>2***; 1>3***; 2>3***
**IMPULSIVENESS**						
Barratt	38.50±10.65	56.76±17.54	41.25±9.63	35.32±7.27	53.14***	1>2***; 1>3***; 2>3***
**ANGER**						
STAXI-State anger	1.44±3.95	7.86±11.22	1.58±2.91	0.68±1.44	35.96***	1>2***; 1>3***
STAXI-Trait anger	6.28±4.49	13.18±5.60	8.86±4.73	4.41±2.59	78.48***	1>2***; 1>3***; 2>3***
STAXI-Anger expression and control	32.81±7.55	35.99±5.87	34.43±8	31.76±7.55	5.20**	**2>3** *

Note. The data refer to the mean ± standard deviation (*SD*).

*p<0.05 **p<0.01 ***p<.001.

High, moderate and low level of violence and psychopathology

**Table 3:** Differences between the three types of batterers regarding the frequency of violence towards the partner.

CTS2 SUBSCALES	Sample (n = 266)	Type I High-level (n = 19)	Type II Moderate-level (n = 74)	Type III Low-level (n = 173)	*F*_(2,263)_	Bonferroni
**PSYCHOLOGYCAL AGGRESSION**						
Minor	15.53±21.81	65.68±27.52	19.85±20.53	8.17±11.60	114.01***	1>2***; 1>3***; 2>3***
Severe	3.68±9.25	19.05±20.89	5.44±10.06	1.24±3.14	44.66***	1>2***; 1>3***; 2>3**
**PHYSICAL AGGRESSION**						
Minor	3.88±8.47	23.37±18.76	3.85±5.15	1.75±3.97	95.78***	1>2***; 1>3***
Severe	0.99±3.32	6.58±10.19	0.83±1.55	0.45±1.16	37.28***	1>2***; 1>3***
**SEXUAL COERCION**						
Minor	1.56±5.04	5.63±8.99	3.09±7.59	0.46±1.48	15.17***	1>3***; 2>3***
Severe	0.22±2.18	2.84±7.86	0.03±0.18	0±0.05	16.66***	1>2***; 1>3***
**INJURY**						
Minor	1.10±4.45	6.42±12.67	1.49±4.30	0.35±1.47	18.44***	1>2***; 1>3***
Severe	0.72±3.42	5.58±9.34	0.94±3.68	0.10±0.34	26.45***	1>2***; 1>3***

Note. Data refer to the average of episodes in the last year of cohabitation with the partner ± standard deviation (*SD*).

*p<0.05 **p<0.01 ***p<.001.

High, moderate and low level of violence and psychopathology

As can be seen in Tables 2 and 3, the differences found indicate that the three groups are different on all these psychological measures, and that there is a continuum from lower to higher levels of psychopathology.

Statistically significant differences were found for the three types of perpetrators of intimate partner violence on all the variables included in Table 2. There were significant differences on alcohol use, borderline personality, antisocial personality, primary and secondary psychopathy, impulsivity and trait anger between the three groups. For each of these variables, the high-level psychopathology group (Type I) evidenced higher levels in comparison with the moderate-level (Type II) and low-level (Type III) groups. Similarly, the moderate-level group evidenced higher levels for all these variables when compared to the low-level group. On the other hand, results related to general physical and psychological aggression indicated that there were only significant differences between the high-level group and the low-level group, as well as between the moderate-level group and the low-level group. Both the high-level (Type I) and the moderate-level group (Type II) had higher levels of general aggression when compared with the low-level group (Type III). However, there were no differences between the high level and moderate-level groups with respect to general physical and psychological aggression. With respect to anger expression and control, significant differences were only observed between the moderate-level group and the low-level group (see Table 2).

With respect to levels of aggression towards the partner, measured with the CTS2, there were again statistically significant differences in all the analysed subscales (see Table 3). The three groups were significantly different on, both minor and severe psychological aggression. Thus, the high-level violence group had a significantly higher average of psychological aggression episodes than the moderate-level group; similarly, the average of the moderate-level violence group was significantly higher than the average of the low-level group. However, only minor differences were found between the high-level group and the other two groups on minor and severe physical aggression, severe sexual coercion, and minor and severe injury (see Table 3).

Operationally, the high-level violence and psychopathology group or Type I (7.1% of the sample) is characterized by higher levels of psychological, physical and sexual aggression and injury to the partner; Type I also shows higher levels of general aggression and psychopathology (borderline and antisocial personality, primary and secondary psychopathy traits, impulsivity, hostility, and trait and state anger), and alcohol use. The moderate-level group or Type II (27.8% of the sample) shows higher levels of psychological aggression towards the partner and lower sexual coercion in comparison with the low-level group or Type III; Type II also shows high levels of general aggression, alcohol use and psychopathology (borderline and antisocial personality characteristics, primary and secondary psychopathy traits, impulsivity, anger, hostility, trait anger, and anger expression and control). Finally, the low-level group or Type III (65% of the sample) shows a lower level in all the variables analysed, either in terms of aggression towards the partner, general violence or presence of psychopathology.

### Evidence for the validity of the clusters

To validate the typology found, we then compared the three clusters of 6 external validation variables: number of arrests prior to pre-treatment assessment, obtained through the Spanish Police electronic database related to intimate partner violence; number of days in the past month (prior to pre-treatment assessment) in which the patients had serious problems with their family, and number of days they had serious problems with other people; years of lifetime consumption of alcohol in large quantities, cocaine, and cannabis. The information about problems with the family and other people and drug use was obtained by means of interview during the pre-treatment assessment.

The results obtained show the existence of statistically significant differences in the three groups of variables analyzed (see Table 4). Type I has a greater number of police pre-arrests than Type II and III, and Type II more than Type III. The same results were obtained with the other variables.

**Table 4:** Validation of the typology found: differences between the three types of batterers in number of prior arrests, family and social variables and psychoactive substance consumption.

	Sample (n = 266)	Type I High-level (n = 19)	Type II Moderate-level (n = 74)	Type III Low-level (n = 173)	*F*_(2,263)_	Bonferroni
**POLICE INFORMATION**						
Prior arrests	0.49±0.56	0.95±0.52	0.58±0.55	0.39±0.55	10.43***	**1>2** * ; 1>3***; 2>3 *
**DAYS IN THE PAST MONTH WITH SERIOUS PROBLEMS**						
Family	0.55±3.17	3.05±7.08	1.11±4.63	0.03±0.18	10***	**1>2** * ; 1>3***; 2>3 *
Others	0.48±2.53	2.89±7.51	0.86±2.66	0.05±0.25	13.09***	1>2**; 1>3***;2>3 *
**YEARS OF LIFETIME CONSUMPTION**						
Alcohol - large quantities	4.45±7.98	11.11±11.73	5.85±9.42	3.12±6.22	10.90***	**1>2** * ; 1>3***; 2>3 *
Cocaine	1.37±4.23	5.05±4.95	2.08±6.08	0.66±2.66	11.56***	**1>2** * ; 1>3***; 2>3 *
Cannabis	2.08±5.13	6.42±7.13	2.96±7.10	1.23±3.27	11.04***	**1>2** * ; 1>3***; 2>3 *

Note. The data refer to the mean ± standard deviation (*SD*).

*p<0.05 **p<0.01 ***p<.001.

High, moderate and low level of violence and psychopathology

These data imply complementary support for the typologies obtained with the psychopathological and aggression variables that were analysed in the cluster analysis. They also sustain the triumvirate obtained, with Type I individuals having a higher level of social deviance (e.g. police arrests, more social and family problems and a higher consumption of alcohol and drugs) than Type II, and Type II higher than Type III.

Lastly, to test the stability of the obtained typology, we carried out a two-step cluster analysis, with the same variables and in the same order in a different sample from the same population, court referred men from the Region of Madrid convicted of a crime of gender violence placed in mandated court diversion program. Approximately 50% were randomly selected, leaving a sample of 228 court mandated perpetrators of intimate partner violence. Results reveal a three cluster solution: one group made up of 8.8% of the sample (n = 20) which corresponds to Type I, one group of 35% (n = 81) or Type II and lastly a group of 55.7% of the sample (n = 127) or Type III. It is important to note that these percentages are similar to those obtained with the sample of 266 patients (7.1%, 27.8% and 65% respectively) which allows us to reach the conclusion that the obtained clusters are relatively stable.

## Discussion

This study confirms the existence of a typology consisting of three batterer subtypes in a criminal justice sample. These results are consistent with those found in previous studies [3]–[6], [11]. The low-level violence and psychopathology group shows lower levels of psychopathology and lower frequency of violence towards the partner. The moderate-level violence and psychopathology group is in between the two groups and, finally, the high-level violence and psychopathology group, which shows a higher level of deviation in the psychopathological characteristics analysed and higher severity and frequency of violence towards the partner. With respect to socio-demographic characteristics of the three groups, the low–level violence group was the oldest, while there was a higher proportion of unemployed people, pensioners and retirees in the high-level violence and psychopathology group. These results are consistent with studies that link increasing age with a decline of aggression towards the partner [32] and other studies that indicate that income level and socioeconomic status are risk factors for abuse [5], [33].

The typology obtained in this study is consistent with that proposed by Cavanaugh and Gelles [11], that compiled the most common typologies of perpetrators of intimate partner violence [3], [4], [12]–[14] and successfully identified similarities across models. The correspondence between the three types of perpetrators proposed by Cavanaugh and Gelles [11] and the typology found in this investigation is shown in Table 5.

**Table 5:** Comparison between batterers’ profiles obtained in this study and Cavanaugh and Gelles [11] proposal.

Cavanaugh and Gelles [11] typology	Description of the typology found in this study
**High risk** *Generally violent/antisocial* [3] *Types I and II* [13] *Antisocial* [4] *Type I* [14] *Intimate terrorist* [15]	**Type I or High-level violence and psychopathology group**In comparison with the other two groups, they show:Higher frequency of minor and severe violence towards the partnerHigher level of general aggressionHeavier alcohol useHigher presence of borderline and antisocial personality characteristicsHigher presence of primary and secondary psychopathy traitsHigher impulsivityGreater anger and hostility
**Moderate risk** *Dysphoric-borderline* [3] *Passive aggressive-dependent* [4] *Type II* [14]	**Type II or Moderate-level violence and psychopathology group**In comparison with the Low-level group, they show:Higher frequency of minor psychological aggression and sexual coercion towards the partnerHigher level of general aggressionHeavier alcohol useHigher presence of borderline and antisocial personality characteristicsHigher presence of primary and secondary psychopathy traitsHigher impulsivityGreater anger and hostility
**Low risk** *Family only* [3] *Type III* [13] *Nonpathological* [4] *Common couple violence* [15]	**Type III or Low-level violence and psychopathology group**They show the lowest levels of:Aggression towards the partner:General aggressionAlcohol useBorderline and antisocial personality characteristicsPrimary and secondary psychopathy traitsImpulsivityAnger and hostility

The present results show evidence favoring heterogeneity and not homogeneity with respect both to type of perpetrator and type of violence [34]. A recent study provides preliminary evidence that attention to offender heterogeneity improves the ability to predict treatment outcome [6]. It may no longer be adequate to conduct studies comparing violent and nonviolent men; instead, it may be more adequate to identify subtypes of batterers and then compare them with each other and with nonviolent comparison groups on variables of theoretical interest. These results also suggest that researchers would benefit from examining how various subtypes of violent men respond to different treatment programs.

The fundamental goal of these programs is to reduce the levels of sexual, psychological and physical violence in order to protect the victims. There is evidence that some of these programs may help these men learn strategies to reduce their violence with subsequent reductions in the probability of recidivism [35], [36]. However, these programs have very high drop out rates [37] which are in turn related to subsequent recidivism rates [38]. It, however, may be possible to improve on the outcomes achieved by these programs by paying attention to the heterogeneity in both the type of violence and the type of perpetrators and tailoring these interventions to address these characteristics [34]. For example, in a post hoc analysis of data from a study comparing cognitive-behavioral-feminist treatment to a new process-psychodynamic treatment designed to help men examine the traumas they have experienced, Saunders [39] found that batterers scoring high on an antisocial measure did better in the structured cognitive-behavioral intervention, whereas batterers scoring high on a measure of dependency did better in the new intervention.

However, despite the clinical usefulness of the development of typologies and all the progress made on this subject, there are some important issues that studies on batterer typologies should address in the future. For example, prospective longitudinal studies are needed to better identify the developmental pathways resulting in different subtypes of aggressive men. Future longitudinal studies should be conducted, examining constructs assumed to predict the use of violence among samples of adolescents or children and then observing the relationship between these variables and the emergence of relationship violence as study participants enter intimate relationships. So far, studies on typologies classify subjects into different categories on the basis of their behavior at one point in time. Furthermore, for example, Cavanaugh and Gelles [11] pointed out that it is unlikely that batterers move from one type to another. However, it appears that empirical evidence points to the opposite direction. For example, Holtzworth-Munroe et al. [40] found that family-only violent men were more likely to desist from violence over time than the other types, while other studies have also found that there are changes over time in the frequency of aggression towards the partner [32].

One of the few studies to analyse the evolution of these typologies over time is the one by Holtzworth-Munroe et al. [8]. They set out to analyse the characteristics of the sample on which they tested their typology [5] 1.5 and 3 years after the initial assessment. The results allowed them to conclude that there is some continuity over time. For example, the *borderline/dysphoric* and *generally violent/antisocial* men still had the highest levels of violence, and the *generally violent/antisocial* men were the least likely to desist from violence.

In future research it would also be important to analyse whether the typologies are really formed by groups that differ qualitatively or, on the contrary, just quantitatively within a single continuum of violence risk. In this regard, Cavanaugh and Gelles [11] proposed their typology, consisting of three types of batterers −low, moderate and high-risk offenders− which are concordant with low, medium and high levels of psychopathology, severity and frequency of violence towards the partner, and criminal history. White and Gondolf [41] also proposed six types of batterers using MCMI scores, and concluded that those six types are actually a continuum ranging from modest personality dysfunction characteristics to a greater severity of the dysfunction. Therefore, some studies point out that the different types of batterers seem to be ordered along a couple of dimensions such as violence and psychopathology, rather than stating that the various types of batterers are qualitatively different. This is an issue that will certainly be analysed in future research.

Finally, it is important to highlight that typologies do not usually include dyadic variables, i.e. variables related to the couple and the type of relationship. However, including such variables in the development of typologies would allow a more precise understanding of violence [42], [43]. Some studies on typologies have considered certain variables of intimate relationships; however, regarding the category family-only batterers proposed by Holtzworth-Munroe and Stuart [3], for example, dyadic factors have not really been studied in depth, even though several studies have shown the importance of analysing these variables. For example, in a longitudinal study, Capaldi et al. [44] found that the men’s violent behavior remained stable over time if they continued with the same partner, whereas these behaviors decreased if the partner changed. Similarly, several studies indicate that discord within the couple and a bad home environment favour the emergence of violent behaviors [45], [46]. However, despite the relevance of these dyadic variables, being able to assess or work with the victim is very difficult, because the priority is always to protect the victim’s integrity and because in many cases there is an order of protection that prevents working with both partners.

In this sense, this study’s main limitation is that of not having information concerning the nature of the relationship from either victims or perpetrators, mainly for family only aggressors, and future research should try to get a more precise description, at least from one member of the dyad. This information could give a more adequate understanding of the dynamic variables that are involved in any relationship and a better way of developing a functional analysis that can help to develop a more effective psychological intervention for both aggressors and victims.

To sum up, despite the different aspects that future research on batterer typologies must address, the study and development of these typologies and the results of the present investigation should be considered in relation to three key issues: a) the identification of differences and consistencies in the violent behavior shown by batterers will help determine underlying processes that contribute to violence in the family, together with causes and consequences. The resulting typologies will provide practitioners with risk characteristics, aiding the process of risk assessment; b) a classification system will aid treatment evaluation and encourage the development of ‘best practice’ treatment programs that will be more effective in preventing further victimization; c) the capacity of police, social service and health sectors to deal with domestic violence can be improved by tailoring interventions towards specific types of offenders.
